# Rosmarinic acid alleviates intestinal inflammatory damage and inhibits endoplasmic reticulum stress and smooth muscle contraction abnormalities in intestinal tissues by regulating gut microbiota

**DOI:** 10.1128/spectrum.01914-23

**Published:** 2023-08-18

**Authors:** Kan Li, Jiawei Wu, Shuang Xu, Xueying Li, Yanhe Zhang, Xue-jiao Gao

**Affiliations:** 1 College of Veterinary Medicine, Northeast Agricultural University, Harbin, Heilongjiang Province, China; 2 Heilongjiang Key Laboratory for Laboratory Animals and Comparative Medicine, Northeast Agricultural University, Harbin, China; Jilin University, Changchun, China

**Keywords:** inflammatory bowel disease, host–bacterial interactions, gut microbiota, smooth muscle contraction

## Abstract

The host–bacterial interactions play the key role in inflammatory bowel disease (IBD). Dysbiosis of the intestinal flora can lead to pathological changes in the intestine. Rosmarinic acid (RA) is a natural phenolic acid compound with antioxidant, anti-cancer, anti-inflammatory, anti-apoptotic, anti-fibrotic, and anti-bacterial activities that has a palliative effect on acute IBD. We have established an *in vivo* model for mice. Histological staining was performed to directly observe RA alterations in the intestinal tract. The alteration of RA on mouse intestinal flora was observed by 16S rRNA high-throughput sequencing, and the effect of RA on intestinal mechanism of action was detected by qPCR and western blot. The results showed that RA had a significant protective effect on the intestine. RA upregulated the abundance of *Lactobacillus johnsonii* and *Candidatus Arthromitus sp SFB-mouse-NL* and downregulated the abundance of *Bifidobacterium pseudolongum*, *Escherichia coli,* and *Romboutsia ilealis*. RA downregulated the expressions of ROCK, RhoA, CaM, MLC, MLCK, ZEB1, ZO-1, ZO-2, occludin, E-cadherin, IL-1β, IL-6, TNF-α, GRP78, PERK, IRE1, ATF6, CHOP, Caspase12, Caspase9, Caspase3, Bax, Cytc, RIPK1, RIPK3, MLKL, and upregulated the expression of IL-10 and Bcl-2. These results displayed that RA inhibited the inflammation, which is caused by tight junction damage, by repairing intestinal flora dysbiosis, relieved endoplasmic reticulum stress, inhibited cell death, and corrected smooth muscle contractile dysregulation. The results of this study revealed RA could have a protective effect on the small intestine of mice by regulating intestinal flora.

**IMPORTANCE:**

Inflammatory bowel disease (IBD) is a chronic, relapsing, remitting disorder of the gastrointestinal system. In this study, we investigated the protective effects of rosmarinic acid on the intestinal tract. The results showed that RA was effective in reducing inflammatory damage, endoplasmic reticulum stress, smooth muscle contraction abnormalities, and regulating intestinal flora disorders.

## INTRODUCTION

Inflammatory bowel disease (IBD) is a chronic, relapsing, remitting systemic gastrointestinal tract disease with a lifelong inflammatory process that can lead to death in severe cases (1). The underlying cause of IBD has not yet been identified. This heterogeneous disease involves a complex interaction with genetic variability, the host immune system, and environmental factors (2). Moreover, the alteration of intestinal flora was found to play a crucial role in triggering chronic inflammation. The IBD is mostly associated with impaired host mucosal barrier function involving in host–microbiome interactions (3). The intestinal tight junction proteins are an important part of the intestinal barrier formation (4). IBD also affects the contraction of smooth muscle in the intestine (5). The intersection of endoplasmic reticulum stress (ERS) with multiple inflammatory pathways can initiate and exacerbate chronic disease, leading to cell death (6, 7). Intestinal flora disorders are closely related to intestinal inflammation development. The maintenance of intestinal flora homeostasis is a potential treatment for IBD.

The development of intestinal flora was in parallel with the growth and development of the host. Its temporal stability and diversity are maintained from adulthood until death (8). The human gut contains up to 100 trillion microorganisms. And up to 40,000 species of bacteria in at least 1,800 genera have been identified in these intestinal florae, with 100 times the genetic content of humans (9). The intestinal flora is a complex microbial ecosystem, representing a mutually beneficial symbiosis between the intestinal microbiota and the host. It plays a crucial role in maintaining the maturation and function of the host immune system. It makes an important contribution to the maintenance of the internal homeostasis of the host (10 – 13). The previous studies have shown that intestinal flora played an important role in regulating chronic diseases including IBD, obesity, type 2 diabetes, cardiovascular disease, cancer, and neurodegenerative diseases (14 – 18). Appropriate intestinal colonization stimulates maturation of lymphoid tissue associated with the intestinal mucosa by specific microbial communities during host primordial stages (19). If the proper intestinal flora is not developed during this life stage and the intestinal immune barrier is not perfected, the function of intestinal immune system will be impaired, leading to an increase in the incidence of certain intestinal diseases (20, 21). Intestinal flora is essential for maintaining intestinal homeostasis. The flora disturbance could alter the structure and function of immune system, reshape the immune microenvironment, and promote the development of specific diseases (22).

Smooth muscle was also shown the important function on maintaining homeostasis of body functions and responding adaptively to stress imposed by pathological disorders (23). It has reported the homeostasis of intestinal flora was closely related to the normal peristalsis of the gastrointestinal tract. The alteration of intestinal flora induced an abnormal contraction of intestinal smooth muscle (24). The smooth muscle contraction occurs in two main pathways. One is the calcium-dependent pathway, which the Ca^2+^ binds to the calmodulin (CaM) that regulates myosin filaments (smooth muscle) on actin thin filaments (in transverse muscle), and then CaM interacts with myosin light chain kinase (MLCK) to phosphorylate myosin light chain (MLC) (25). The other is a non-calcium-dependent pathway associated with signaling molecules such as RhoA, MLCK, and MLC (26, 27).

Natural polyphenol acid was considered as a regulator of nutritional metabolism and metabolic diseases with their anti-inflammatory and antioxidant functions. The natural polyphenol acid was proved to maintain a balance between intestinal microbes and their hosts (28). Rosmarinic acid (RA) is a naturally occurring phenolic acid compound that is widely found in a variety of plants (29). RA is one of the most attractive phytochemicals due to its significant pharmacological activity(30), such as antioxidant, anti-cancer, anti-inflammatory, anti-apoptotic, and anti-fibrotic (31 – 34). The previous research had shown RA have a palliative effect on a variety of diseases such as acute inflammatory bowel disease, neurological disorders, non-alcoholic fatty liver disease, etc. (35 – 37), but the underlying mechanism of action is unclear. The present study aimed to explore the effect of acid on intestinal inflammation by regulating intestinal flora. The gut microbiota was sequenced in a mice model tight junction dysregulation, abnormal smooth muscle contraction, endoplasmic reticulum stress, and cell death.

## RESULTS

### RA alleviates DSS-induced inflammatory bowel disease symptoms

**Fig 1:**
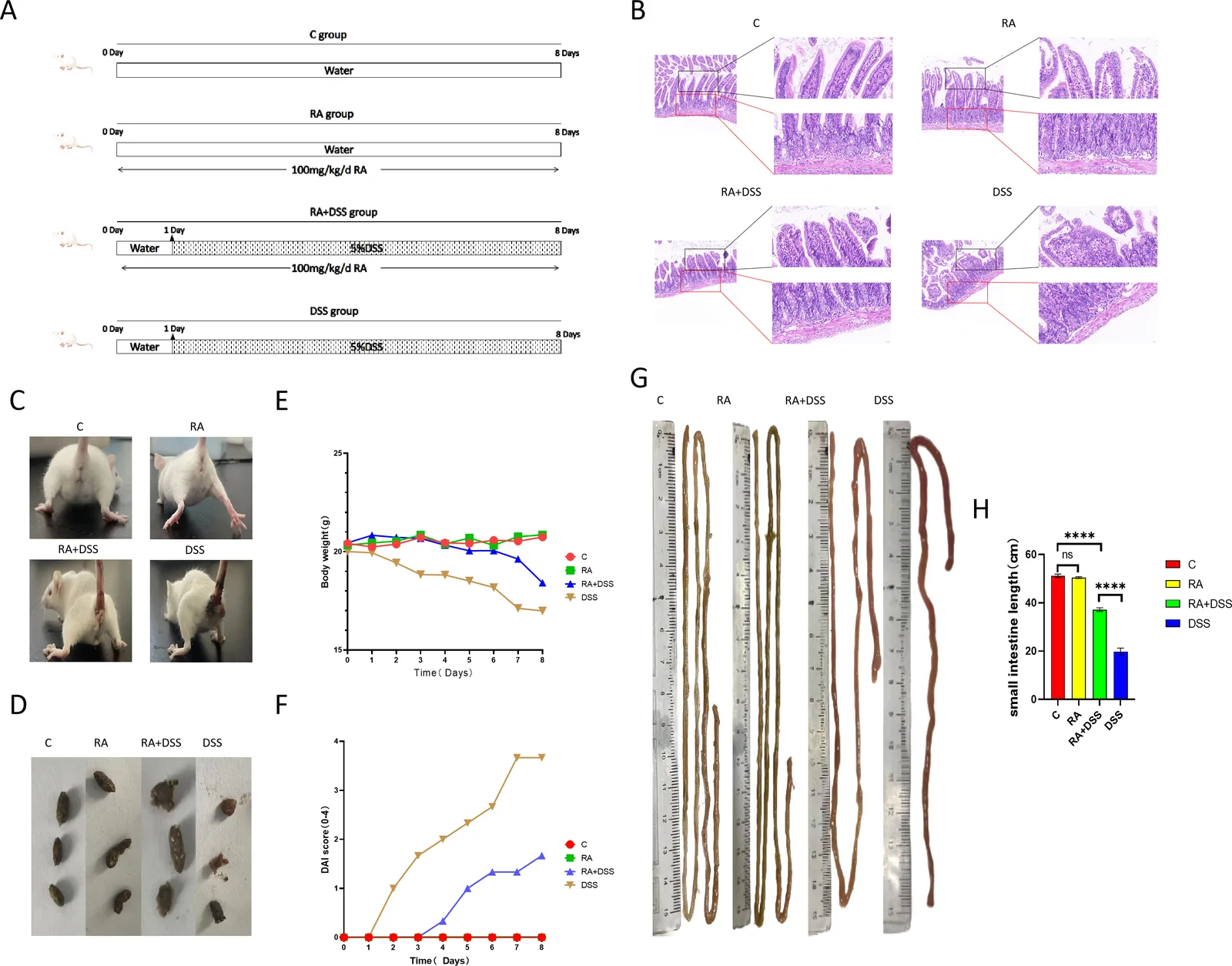
RA alleviated the inflammatory symptoms caused by DSS. (**A**) The graphical representation of the study design. RA gavage for 8 days in the RA group and RA +DSS group, DSS gavage for 7 days in the RA + DSS group after 1 day of RA gavage, and normal water intake on the first day in the DSS group, followed by switching to DSS gavage for 7 days to induce small bowel damage; (**B**) HE stained; (**C**) defecation of mice in each group; (**D**) fecal status of mice in each group; (**E**) bodyweight monitor every day; (**F**) DAI score monitor every day; (**G**) effect of RA and DSS on the length of the small intestine in mice; (**H**) small intestine length. Data were expressed as means ± SEM (*n* = 4). *P < 0.05; **P < 0.01. ***P < 0.001, ****P < 0.0001.

The mice all showed obvious signs of IBD after receiving dextran sulfate sodium salt (DSS), and histological examination showed that DSS significantly reduced height or even breakage of small intestinal villi, severe deformation of cup cells, absence of muscle-arranged cells, and inflammatory infiltration compared with control group (CG). In contrast, RA showed significant protection against DSS-induced small intestinal injury with intact morphology of epithelial cells, reduced cupular cell deformation, and abundant muscle-arranged cells, but still with a small infiltration of inflammatory cells (Fig. 1B). The RA also relieved the severe weight loss, diarrhea, blood in the stool, and small bowel length shortening phenomena (Fig. 1G and H) induced by DSS. It was quantifiable comparison with exhibiting severe disease activity index (DAI) scores (Fig. 1C and F).

### The regulation of RA on intestinal flora discrepancy

**Fig 2:**
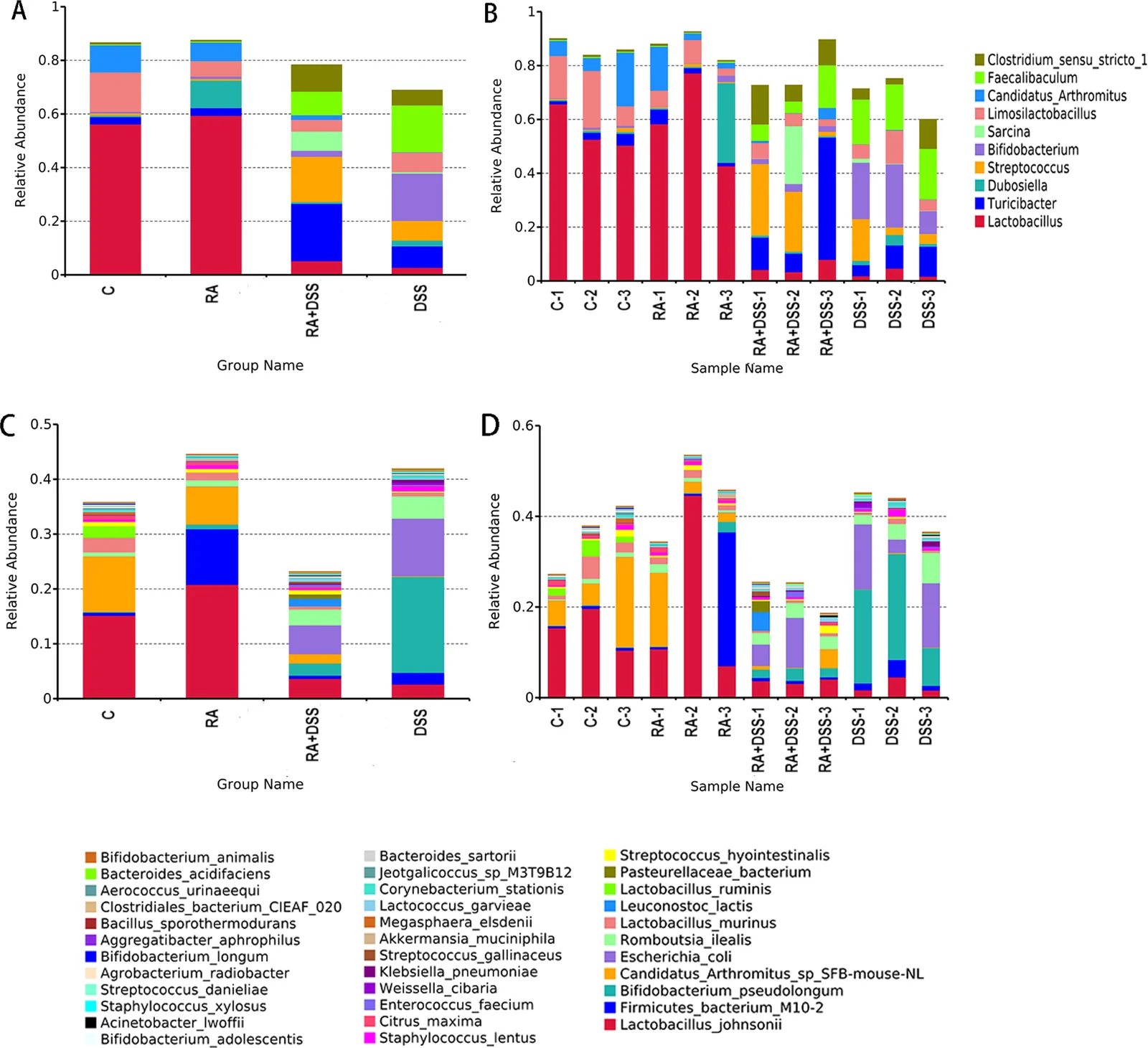
Intestinal flora species and ratios in mice. (**A**) Histogram of relative abundance of sample species (top 10, genus); (**B**) histogram of relative abundance of grouped species (top 10, genus); (**C**) histogram of relative abundance of sample species (top 35, species); (**D**) histogram of relative abundance of grouped species (top 35, species).

The metagenomic technology was used to analyze the composition of intestinal flora in each group. Figure 2A and B showed the species composition analysis of all samples at the genus level. It showed a significant change in species composition between the groups. The bacterial genera with the highest abundance values in CG were *Lactobacillus* (56.2679%), *Limosilactobacillus* (14.7067%), and *Candidatus Arthromitus* (10.0929%). In group RA, the bacterial genera with the highest abundance values were *Lactobacillus* (59.4357%), *Dubosiella* (10.166%), and *Candidatus Arthromitus* (6.9152%). The genera with the highest abundance values were *Turicibacter* (21.432%), *Streptococcus* (16.9104%), and *Clostridium sensu stricto1* (9.8684%) in the RA + DSS group. In the DSS group, they were *Bifidobacterium* (17.6307%), *Faecalibaculum* (17.4426%), and *Turicibacter* (7.9732%). Figure 2C and D showed that *Limosilactobacillus* had the highest abundance values in CG among these four groups. *Dubosiella* and *Lactobacillus* in RA group had higher abundance values than other groups. In the RA + DSS group, the abundance values of *Clostridium sensu stricto1*, *Sarcina*, *Streptococcus*, and *Turicibacter* were higher than those in other groups. In the DSS group, the abundance values of *Bifidobacterium* and *Faecalibaculum* were higher than other groups, and the abundance values of *Lactobacillus* were lower than the other three groups. It can be found that the species abundance values of *Bifidobacterium pseudolongum*, *Escherichia coli*, and *Romboutsia ilealis* are higher in CG than in the other three groups. The abundance of *Lactobacillus johnsonii* and *Candidatus Arthromitus sp SFB-mouse-NL* was the lowest among these four groups.

### Effect of RA on the abundance and diversity of intestinal flora

**Fig 3:**
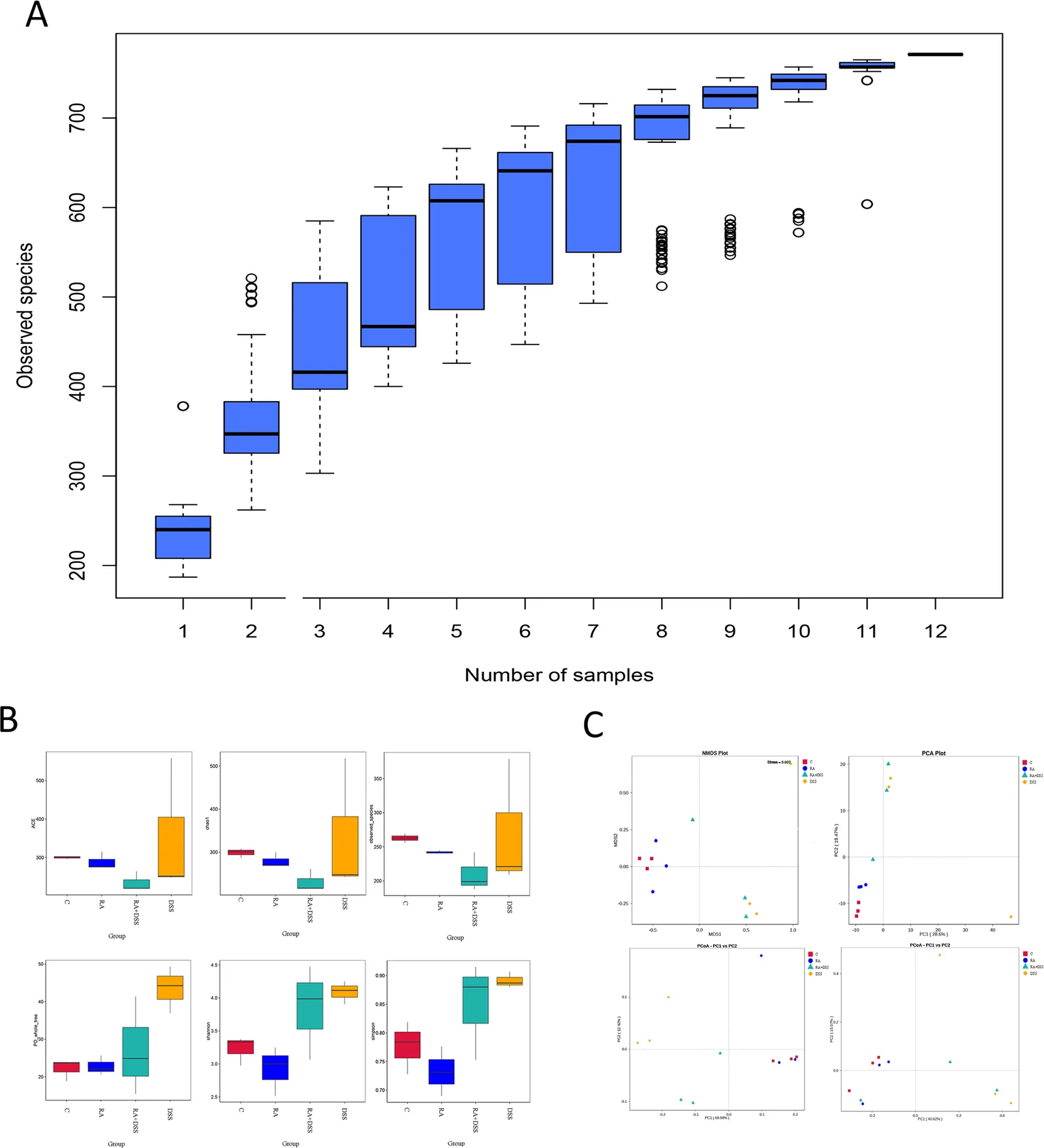
Analysis of the diversity and abundance of small intestinal contents in each group. (**A**) Species accumulation box plot: horizontal coordinates indicate the sample size and vertical coordinates indicate the number of OTUs (operational taxonomic units) after sampling; (**B**) alpha diversity indices of fecal samples in each group; (**C**) dimensionality reduction analysis of intestinal microorganisms in various groups of mice.

As the sample size increased, a large number of species were found. The trend of species was found to gradually plateau, indicating that the collected fecal samples were sufficient for data analysis (Fig. 3A). The abundance values of intestinal flora were calculated with ACE, Chao, observed species, phylogenetic diversity index with PD whole tree, and diversity of flora with Shannon, Simpson. It was found that the abundance values of flora were different in all groups, and compared to the CG, the DSS group presented increased species diversity and evolutionary changes. The RA + DSS group showed a decrease in species diversity, evolutionary diversity, and richness compared to the DSS group (Fig. 3B). PCoA analysis (principal coordinates analysis) was performed with Weighted Unifrac distance and Unweighted Unifrac distance. The PCA (principal component analysis) analysis and NMDS (nonmetric multidimensional scaling) analysis were to analyze the differences in gut flora composition between groups. The results found that the intergroup difference between CG and RA was small, the intergroup difference between DSS and CG was the largest, and the intergroup gap between the RA + DSS group and group C was smaller than that between DSS and CG (Fig. 3C).

### Species differences in RA-induced DSS-induced small intestinal flora

**Fig 4:**
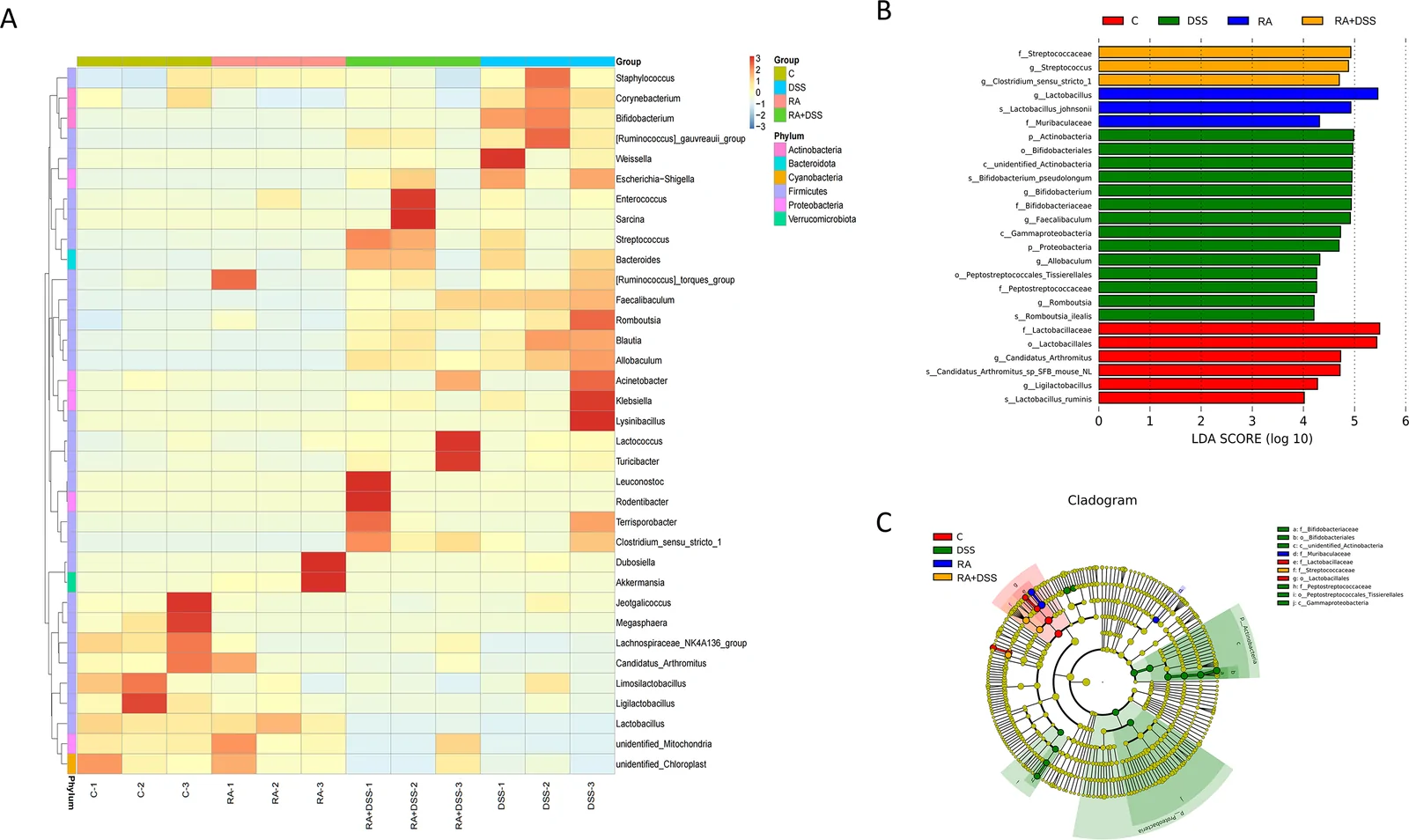
Species difference analysis and marker species. (**A**) Species abundance clustering heat map; (**B**) histogram of LDA value distribution; (**C**) LEfSe evolutionary branch diagram.

Based on the species annotations and abundance information at the genus level, the top 35 species at the genus level in terms of abundance were selected. The species assemblages were found to be different for each group (Fig. 4A). LEfSe (LDA (Linear discriminant analysis) Effect Size effect size) could demonstrate species with significant variability in abundance between groups and also be used to identify marker species in the sample bacterial community between groups. The marker species in each group, with LDA effect size >4 and *P* < 0.05, are shown in Fig. 4B and C. At the genus level, the marker species were *Ligilactobacillus* and *Candidatus Arthromitus* in CG, *Lactobacillus* in RA group, and *Streptococcus* in group RA + DSS. The iconic species of the DSS group were *Bifidobacterium*, *Faecalibaculum*, *Allobaculum*, and *Romboutsia*. At the species level, the iconic species of the CG were *Candidatus Arthromitus sp SFB mouse NL* and *Lactobacillus ruminis*, the iconic species of the RA group was *Lactobacillus johnsonii*, the iconic species of the RA + DSS group was *Clostridium sensu stricto1*, and the iconic species of the DSS group was *Bifidobacterium pseudolongum*.

### Key action target of RA with small intestinal flora

**Fig 5:**
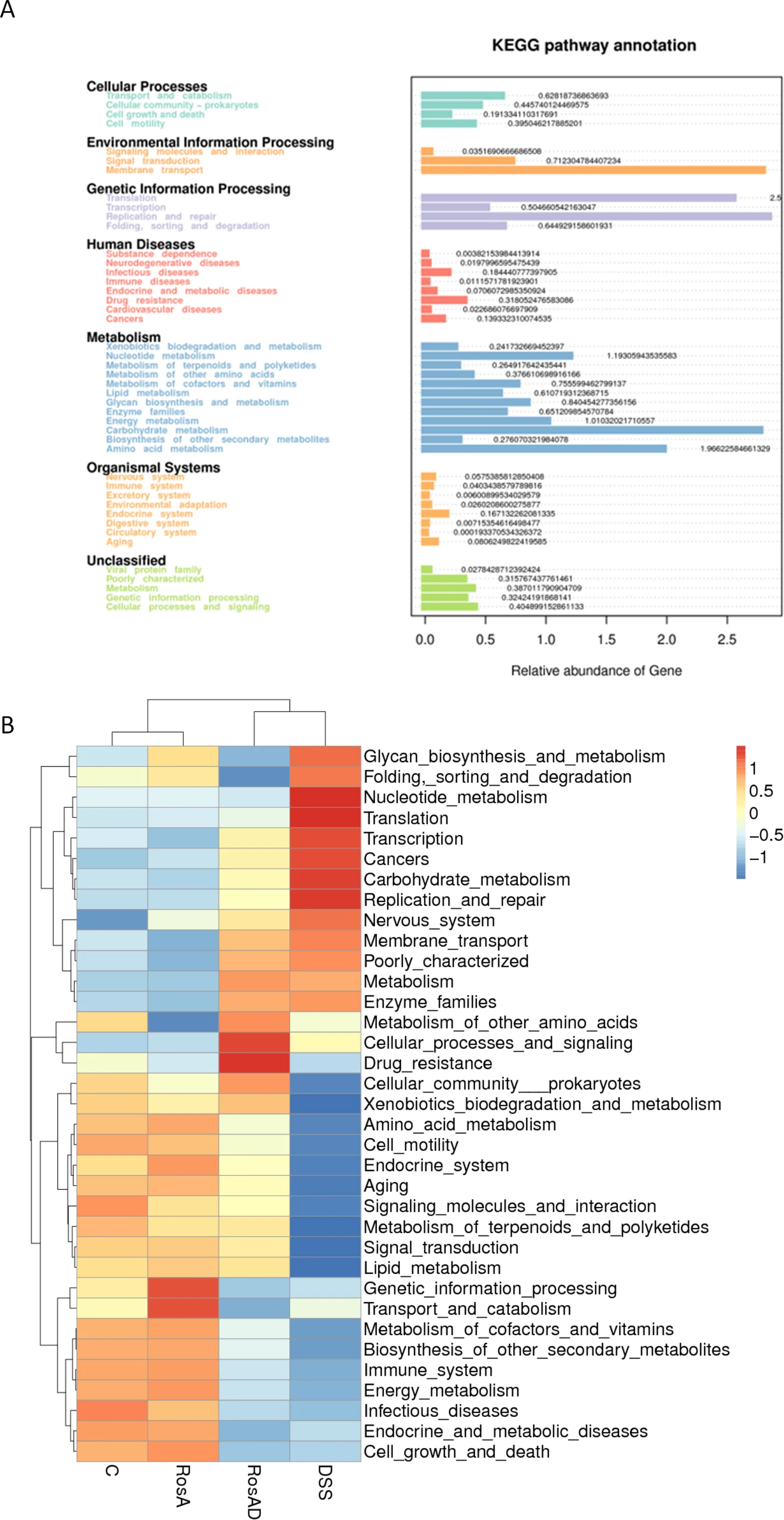
Altered metabolic pathway function. (**A**) Heat map of secondary functional relative horizontal clustering with sample information in the vertical direction and functional annotation information in the horizontal direction; (**B**) predicted KEGG secondary functional pathways.

Through extracting prokaryotic genome-wide 16S rRNA gene sequences from the KEGG database and comparing them with the SILVA SSU Ref NR database, a matrix was established to cluster OTUs (Operational Taxonomic Units) using the SILVA database sequences as reference sequences and then obtain functional information. Figure 5A showed that the abundance values of species associated with Membrane transport, Replication and repair, and Carbohydrate metabolism functions occupied the top three species abundance values. Figure 5B showed that the abundance values of species related to these three functions were significantly upregulated in the DSS group, and the related species in the RA + DSS group were significantly decreased compared to the DSS group.

### RA reduced the injury by DSS induced in small intestinal flora

**Fig 6:**
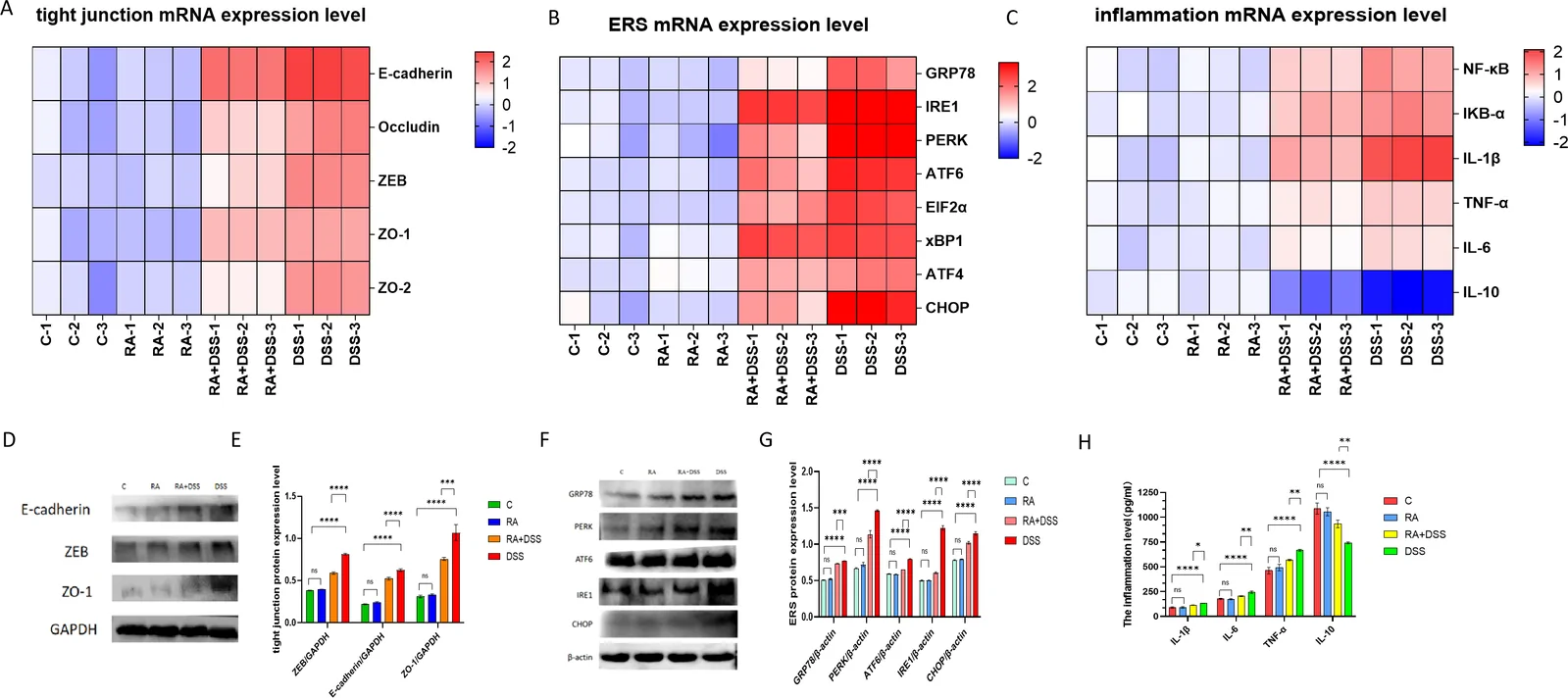
Effects of RA on tight junction damage, inflammation, and endoplasmic reticulum pressure of DSS-molded mice. (**A**) Heat map of relative expression of tight junction mRNA in mouse small intestine; (**B**) heat map of relative expression of inflammation mRNA in mouse small intestine; (**C**) heat map of relative expression of ERS mRNA in mouse small intestine; (**D**) western blot assay of E-cadherin, ZEB, ZO-1; (**E**) protein expression of E-cadherin, ZEB, ZO-1 in RA- and DSS-treated mouse models; (**F**) assay of interleukin-1β (IL-1β), IL-6, IL-10, and tumor necrosis factor-α (TNF-α) in RA- and DSS-treated mouse small intestinal models using enzyme-linked immunosorbent assay (ELISA) kits; (**G**) western blot assay of GRP78, IRE1, PERK, ATF6, EIF2α, xBP1, ATF4, CHOP; (**H**) protein expression of GRP78, PERK, ATF6, IRE1, CHOP in RA- and DSS-treated mouse models. The data are represented as SEM ± mean (*n* = 4) and analyzed using one-way ANOVA and Tukey post-mortality. Control group (**C**), rosmarinic acid treatment group (RA), rosmarinic acid with DSS treatment group (RA + DSS), DSS-induced enteritis (DSS) group (ns, not statistically significant, *P < 0.05, **P < 0.01, ***P < 0.001, ****P < 0.0001).

The protective effect of RA on tissue by regulating intestinal flora was verified with the regulation on tight junction damage, inflammation, and endoplasmic reticulum stress. Figure 6A showed the mRNA levels of E-cadherin, Occlindin, ZO-1, ZO-2, and ZEB were highest in the DSS group than the other groups. The mRNA levels of these genes were significantly lower in the RA + DSS group compared to DSS group. The mRNA levels of inflammatory factors, NF-κB, IκBα, IL-6, IL-1β, and TNF-α were increased in DSS group, except for the IL-10 with the RA treatment. The mRNA levels of inflammatory factors were significantly decreased, but IL-10 was significantly increased compared with the DSS group. Figure 6C shows that the mRNA expression levels of GRP78, IRE1, PERK, ATF6, and CHOP are highest in the DSS group compared with other groups. The mRNA expression levels of GRP78, IRE1, PERK, ATF6, and CHOP were significantly decreased in the RA + DSS group compared with the DSS group. Figure 6D and E shows that E-cadherin, ZO-1, and ZEB had the highest protein levels in the DSS group. The expression of related proteins was significantly decreased in the RA + DSS group compared with the DSS group. The expression of related genes and proteins was not significant in CG and RA groups. To assess the amount of small intestinal inflammation caused by DSS, Elisa kits were used to detect the expression levels of IL-6, IL-10, IL-1β, and TNF-α, which are inflammatory factors. In Figure 6F, the expressions of inflammatory factors were the highest and the IL-10 was the lowest in the RA + DSS group compared with the DSS group. The expressions of IL-6, IL-1β, and TNF-α decreased and IL-10 increased in the RA + DSS group compared with the DSS group, and the differences in the expression of related genes and inflammatory factors between the C and RA groups were not significant. The differences in the expression of related genes and inflammatory factors between the C and RA groups were not significant. Figure 6G and H showed the highest protein expression levels of GRP78, IRE1, PERK, ATF6, elF2α, xBP1, ATF4, and CHOP in the DSS group. The related protein expression levels protein expression was significantly decreased compared to the RA + DSS group.

### RA reduces DSS-induced cell death in mouse intestinal

**Fig 7:**
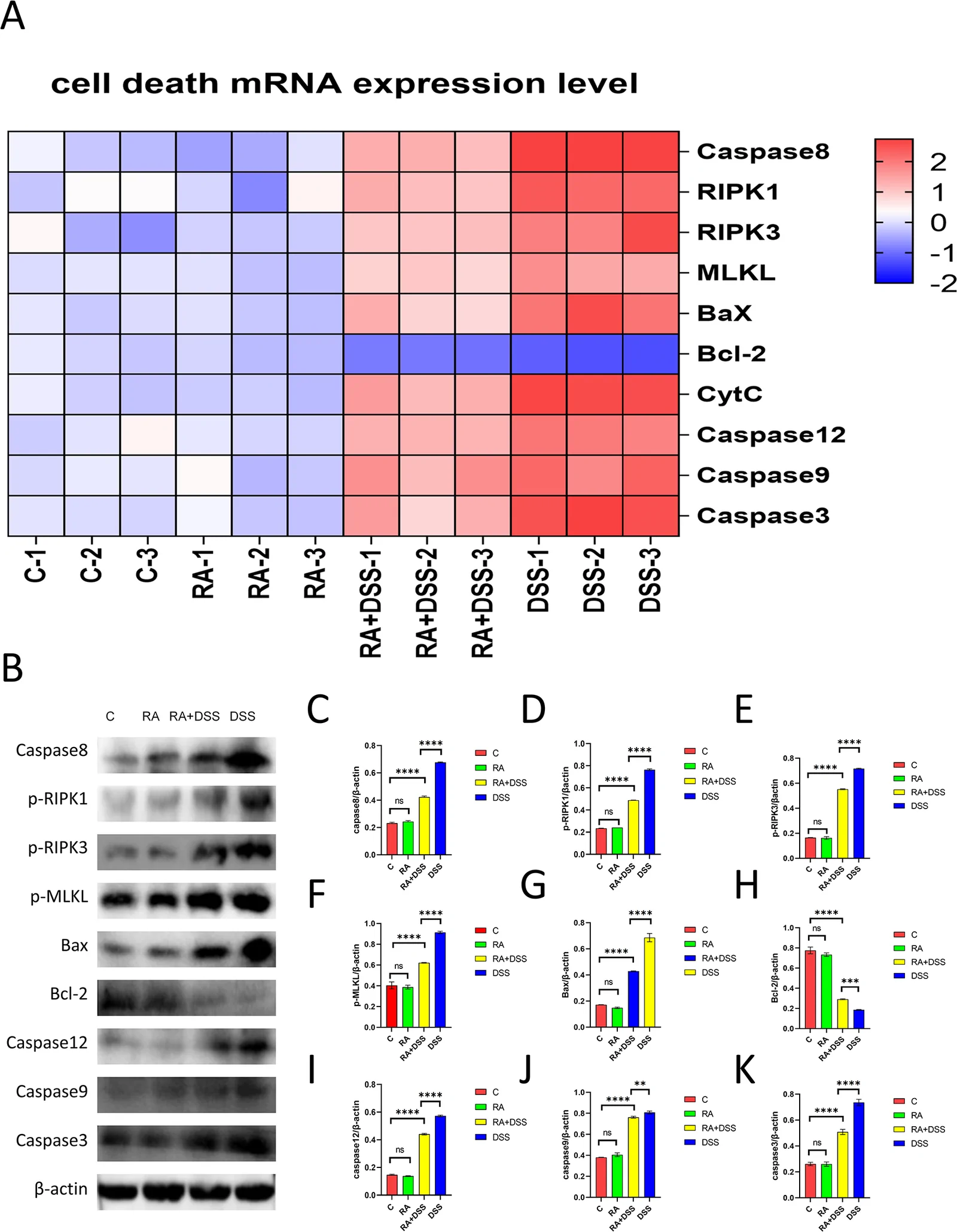
Effects of RA on cell death of DSS-molded mice. (**A**) Heat map of relative expression of cell death mRNA in the small intestine of a mouse; (**B**) western blot assay of Caspase8, *p*-RIPK1, *p*-RIPK3, *p*-MLKL, Bax, Bcl-2, Caspase12, Caspase9, Caspase3; (**C-K**) protein expression of Caspase8 (**C**), *p*-RIPK1(D), *p*-RIPK3 (E), *p*-MLKL(F), Bax(G), Bcl-2(H), Caspase12(I), Caspase9(J), Caspase3(K) in RA- and DSS-treated mouse models. The data are represented as SEM ± mean (*n* = 4) and analyzed using one-way ANOVA and Tukey post-mortality. Control group (**C**), rosmarinic acid treatment group (RA), rosmarinic acid with DSS treatment group (RA + DSS), DSS-induced enteritis (DSS) group (ns, not statistically significant, *P < 0.05, **P < 0.01, ***P < 0.001, ****P < 0.0001).

It was shown that in the DSS group, the mRNA levels of Caspase8, RIPK1, RIPK3, MLKL, Bax, Cytc, Caspase12, Caspase9, and Caspase3 were the highest, and the expression levels of related genes were significantly decreased in the RA + DSS group compared with the DSS group. Meanwhile, the expression of the Bcl-2 gene was the lowest in the DSS group, while the level of the Bcl-2 gene was significantly higher in the RA + DSS group compared with the DSS group (Fig. 7A). The protein expressions of Caspase8, *p*-RIPK1, *p*-RIPK3, *p*-MLKL, Bax, Bcl-2, Caspase12, Caspase9, and Caspase3 were the highest in the DSS group, and the expression levels of related proteins were significantly lower in the RA + DSS group than in the DSS group. The DSS group had the Bcl-2. The protein expression levels of Bcl-2 in the DSS group were the lowest, while the Bcl-2 protein levels in the RA + DSS group were significantly higher than those in the DSS group. There were no significant differences in the expression of related genes and proteins between the C and RA groups (Fig. 7B through K).

### RA reduces the abnormal contraction of a small intestinal smooth muscle caused by DSS in mice

**Fig 8:**
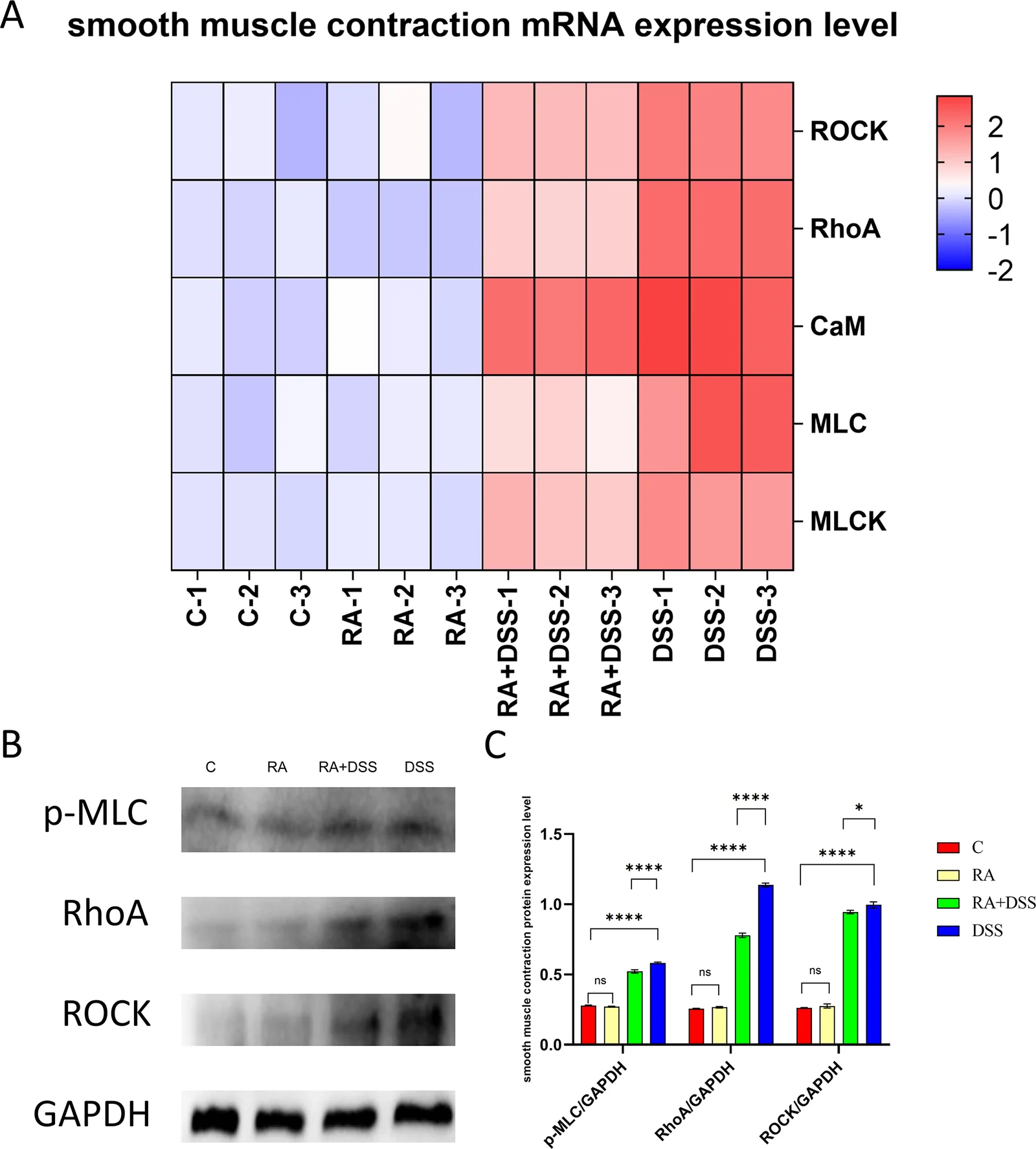
Effects of RA on smooth muscle contraction of DSS-molded mice. (**A**) Heat map of relative expression of smooth muscle contraction mRNA in mouse small intestine; (**B**) western blot assay of *p*-MLC, RHOA, ROCK; (**C**) protein expression of *p*-MLC, RHOA, ROCK in RA- and DSS-treated mouse models. The data are represented as SEM ± mean (*n* = 4) and analyzed using one-way ANOVA and Tukey post-mortality. Control group (**C**), rosmarinic acid treatment group (RA), rosmarinic acid with DSS treatment group (RA + DSS), DSS-induced enteritis (DSS) group (ns, not statistically significant, *P < 0.05, **P < 0.01, ***P < 0.001, ****P < 0.0001).

DSS caused the abnormal contraction of small intestinal smooth muscle, and we examined the gene expression of CaM, MLC, MLCK, RhoA, and ROCK and the protein expression of *p*-MLC, RhoA, and ROCK. Figure 8A indicated that the mRNA levels of CaM, MLC, MLCK, RhoA, and ROCK were significantly increased in the DSS group compared with the other groups. In Fig. 8B and C, compared with the DSS group, the protein expressions of *p*-MLC, RhoA, and ROCK were significantly decreased in the RA + DSS group. There were no significant differences in the expression of related genes and proteins between group C and RA. It indicated that RA could inhibit the DSS-induced abnormal smooth muscle contraction in the mouse intestine.

## DISCUSSION

The balance of intestinal flora contributes significantly to the maintenance of small intestinal health. It has been reported that intestinal flora is part of the intestinal immune barrier and has a role in maintaining the function of the immune system (12, 13). Several studies have found a protective effect on the small intestine through the consumption of natural plant extracts that regulate intestinal flora (38 – 40). RA is an excellent natural antioxidant (29). However, it is not clear whether RA can have a protective effect on the gut by modulating the intestinal flora.

To further investigate the effect of RA on the regulation of intestinal flora in mice, we extracted small intestinal contents for a 16-s high-throughput sequencing analysis. The results showed that the distribution of species in the C and RA groups were similar, and it was found that RA did not cause damage to the intestinal flora of mice, while the diversity and composition of species in the DSS-treated flora changed significantly, and the diversity and composition of species in the RA + DSS group did not change significantly in the DSS group. At the species level, RA could maintain the balance of intestinal flora by reducing the abundance of *Bifidobacterium pseudolongum*, *Escherichia coli*, and *Romboutsia ilealis*, and increasing the abundance of *Lactobacillus johnsonii* and *Candidatus Arthromitus sp SFB-mouse-NL. Lactobacillus johnsonii* is able to reduce inflammation and endoplasmic reticulum stress in mice (41). *Candidatus Arthromitus sp SFB-mouse-NL* plays an important role in the innate and adaptive immune function of the intestine (42). *Escherichia coli* has been reported to be the main cause of acute diarrhea in infants in Brazil (43). *Romboutsia ilealis* is a potentially harmful bacteria in the gut (44). It is worth noting that DSS-induced inflammatory enteritis can lead to damage to the intestinal mucosal barrier. *Bifidobacterium pseudolongum* has a protective effect on the intestinal barrier in mice (45). This suggests that under the body’s self-regulation, the body can repair the intestinal mucosal barrier by increasing the abundance of *Bifidobacterium pseudolongum*. The results showed that RA can reduce intestinal damage caused by DSS intestinal flora disorder in mice by regulating harmful and beneficial bacteria in intestinal flora.

Our study showed that the DSS group caused significant pathological changes in the small intestine, mucosal edema, goblet cell depletion, severe damage to crypts, and inflammatory cell infiltration into the mucosa; RA can alleviate the symptoms caused by DSS; and RA did not cause significant pathological changes in the small intestine compared with the C group. E-cadherin, ZEB, ZO-1, ZO-2, occludin upregulated from the gene level, tightly linked landmark proteins E-cadherin, ZEB, ZO-1 upregulated, tight junction-related genes and abnormal expression of proteins indicate disruption of the intestinal barrier in mice. Tight junction dysregulation is associated with intestinal inflammation. It has been reported that E-cadherin is locally upregulated in inflamed intestinal mucosa (46). Inflammatory cytokines TNF-α and IL-6 can activate the expression of ZEB (47). In inflammatory bowel disease, the expression of ZO-1 can be regulated through the NF-κB pathway (48). IL-1β, IL-6, TNF-α are inflammatory factors, and IL-10 is anti-inflammatory factor (49 – 52). IL-1β, IL-6, and TNF-α levels in the DSS group were increased, anti-inflammatory factor IL-10 levels were reduced. The inflammatory responses are caused by NF-κB/IKB-α classical pathways (53). ERS can intersect with multiple inflammatory pathways and play a role in causing or aggravating disease (6). Inflammatory factors stimulate the production of stress in the endoplasmic reticulum, which leads to the occurrence of endoplasmic reticulum stress (54). GRP78 is a highly conserved protein that is important for maintaining normal cell activity, and the GRP78 gene upregulates PERK, IRE1, and ATF6, which can activate endoplasmic reticulum stress, and ultimately upregulates the ERS transcription factor CHOP, leading to cell damage. RA can protect the mouse small intestine by slowing DSS-induced tight junction dysregulation, inflammation, and endoplasmic reticulum stress through the gut flora.

Pathogenic bacteria produce virulence factors that cause damage to the host by activating cell death pathways (55). TUNEL experiments showed that a large number of small intestinal cells in the DSS group had a large number of deaths, and RA could alleviate the small intestinal phenomenon caused by DSS, and there was no significant change between group C and RA. Through RT-PCR, the mRNA levels of Caspase8, RIPK1, RIPK3, MLKL, Bax, Cytc, Caspase12, Caspase9, Caspase3 increased, and Bcl-2 levels decreased. The protein levels of Caspase8, *p*-RIPK1, *p*-RIPK3, *p*-MLKL, Bax, Bcl-2, Caspase12, Caspase9, and Caspase3 confirmed DSS-induced cell necrosis, apoptosis of the mitochondrial pathway, and apoptosis of the endoplasmic reticulum stress pathway. Caspase8 can inhibit necrotizing apoptosis mediated by RIPK1, RIPK3, and MLKL (56, 57). Caspase8 has long been considered a promoter of apoptosis, capable of inducing mitochondrial apoptosis pathways, and apoptosis cascades (58 – 60). DSS is known to cause endoplasmic reticulum stress in the small intestine of mice. Endoplasmic reticulum stress can induce cascade apoptosis in Caspase12 expression (61). There have been reports of an interaction between two modes of death: apoptosis and cell necrosis (62). The results showed that DSS-induced necrotizing apoptosis, mitochondrial apoptosis, and endoplasmic reticulum stress pathway-mediated apoptosis, and the upregulation of Caspase8 indicated that small intestinal cell death was more likely to be apoptosis. RA can mitigate DSS-induced intestinal death in mice through intestinal flora.

DSS is known to induce inflammatory bowel disease and intestinal inflammation-induced disturbances of the intestinal flora. Disturbances in the intestinal flora can cause abnormal contraction of smooth muscle (53). ROCK is an important regulator of cellular contractility (63). RhoA plays an important role in regulating actin-filament formation and myosin-actin (64). ROCK regulates the phosphorylation of MLCs to regulate the contractility of smooth muscle (65, 66). The results found that five signature gene levels of ROCK, RhoA, CaM, MLC, MLCK, and MLCK smooth muscle contraction in the small intestine of DSS-treated mice were observed to be upregulated, and at the protein level, ROCK, RhoA, *p*-MLC were upregulated. RA slows down smooth muscle contraction by downregulating genes and proteins related to smooth muscle contraction. RA treatment alone cannot affect smooth muscle contraction compared to the RA group. RA is able to mitigate DSS-induced smooth muscle contraction abnormalities by modulating the intestinal flora.

In summary, DSS-induced dysbiosis of the intestinal flora leads to inflammatory damage in the small intestine and to cell death via endoplasmic reticulum stress and mitochondrial pathways, accompanied by abnormal contraction of small intestinal smooth muscle. We found that RA increased the abundance values of *Lactobacillus johnsonii* and *Candidatus Arthromitus sp SFB-mouse-NL* and decreased the *Bifidobacterium pseudolongum*, *Escherichia coli,* and *Romboutsia ilealis* abundance values. The ability of RA to modulate intestinal flora was demonstrated to reduce intestinal damage and alleviate smooth muscle contraction and endoplasmic reticulum stress. Our study enriched the mechanism of DSS-induced enteritis model, while exploring the important role of intestinal flora in small intestinal tissue damage and further validating the protective effect of natural antioxidants on small intestinal tissues. Notably, RA acts as a good natural antioxidant and these findings provide new insights into RA for the treatment of IBD. Understanding the composition of the intestinal flora facilitates the discovery of targeted drugs for the treatment of intestinal diseases, and these data have important implications for the treatment of IBD.

## MATERIALS AND METHODS

### Animals

Forty 4- to 5-wk-old Kunming mice were divided into four groups of 10 mice each. Mice of different sexes were randomly assigned into the four groups. The mice were grown under cyclic light (12 h light, 12 h darkness), temperature 18–22°C, and humidity 50–60% in a naturally ventilated environment. Normal group (CG): the mice were fed with normal food and normal water; RA-treated group (RA): 10 mice were gavaged with rosmarinic acid at 100 mg/kg every 24 h; RA and DSS co-treated group (RA + DSS): the mice were gavaged with RA at 100 mg/kg every 24 h. The drinking water was with 5% DSS; DSS-treated group (DSS): drinking water of mice was with 5% DSS (Fig. 1A). Mice were allowed to eat and drink freely, and after 7 days, mice were euthanized using 100 mg/kg sodium pentobarbital, and samples were quickly taken and stored in a −80°C refrigerator. The present study was approved by the Animal Care and Use Committee of Northeast Agricultural University with the National Research Council’s Guide for the Care and Use of Laboratory Animals.

### Histopathology staining

Three 0.4-cm-long sections of small intestine tissue were cut and fixed in prepared 4% paraformaldehyde, and the tissue was trimmed and dehydrated in xylene and paraffin-embedded overnight. The small intestine was cut into 2-μm-thick slices and soaked in ZGSJ (Masson A) overnight. The small intestine sections were stained with Weggett hematoxylin (Masson A and Masson B in equal amounts) for 1 min, fractionated with 1% acidic ethanol, then stained with scarlet magenta solution (Masson D) for 6 min, fractionated in phosphor-molybdenum-phosphotungstate solution (Masson E) for 1 min, and then transferred directly to aniline blue solution (Masson F) for 2–30 s. Then, it was rinsed briefly in distilled water for 2–5 min. Finally, the slides were decolorized by anhydrous ethanol, clear in xylene, and sealed with a neutral sealant. Sections were scanned with a Pannoramic 250 slide scanner (3D, HITECH). Micrographs were analyzed by the blinded method. Collagen volume was observed by Image Analysis System software (HALO, Indica Labs, American) for collagen volume (CVF, USA). Technical support was provided by Servicebio, Inc. (Wuhan, China).

### Disease activity index (DAI) measures

The DAI values were obtained by observing the rate of weight loss (0 point for no weight loss, 1 point for 1–5% weight loss, 2 points for 5–10% weight loss, 3 points for 10–15% weight loss, and 4 points for >15% wt loss), fecal consistency (0 point for normal fecal consistency, 2 points for loose feces, and 4 points for diarrhea), and fecal bleeding (0 point for no fecal bleeding, 2 points for positive fecal occult blood, and 4 points for dominant fecal bleeding) in each group, and the average of the total scores of the three results was taken.

### Quantitative PCR

To assess mRNA expression levels using RT-PCR, total RNA was extracted from frozen intestinal tissues at 0.1 g from a refrigerator at −80°C. The concentration and purity of the RNA solution were determined by UV spectrophotometry at 260 nm and 280 nm. This was followed by reverse transcription to the cDNA template for RT-PCR assay. For each gene to be measured, the cDNA templates expressing the gene and the sample cDNA were selected for the PCR reaction. Forty cycles were repeated at 94°C for 30 s, 94°C for 5 s, and 60°C for 30 s. Each experiment was repeated three times and each sample was repeated three times. β-actin was used as an endogenous internal standard control. Primers were purchased from Shanghai Triangle Biotechnology. Primers used for qPCR in Table 1 has been in the right place.

**TABLE 1:** Primers used for qPCR

Gene	Primer sequence (5′−3′)
E-cadherin	Forward: 5′- ACCAGCAGTTCGTTGTCGTCAC-3′
	Reverse: 5′- GTTCCTCGTTCTCCACTCTCACATG-3′
ZO-1	Forward: 5′- AACCCGAAACTGATGCTGTGGATAG −3'
	Reverse: 5′- CGCCCTTGGAATGTATGTGGAGAG −3′
ZO-2	Forward: 5′- CATGTCTCTAACGGATGCTCGGAAG −3′
	Reverse: 5′- GTTTAGGGCTGGGATGTTGATGAGG −3′
Occludin	Forward: 5′- TGGAGGCTATGGCTATGGCTATGG-3′
	Reverse: 5′-TTACTAAGGAAGCGATGAAGCAGAAGG-3′
ZEB	Forward: 5′- AAGCCATACGAATGCCCGAACTG-3′
	Reverse: 5′- GCGAGGAACACTGAGATGTCTTGAG-3′
NF-κB	Forward: 5′-CCATAGCCATAGTTGCGGTCCTTC-3′
	Reverse: 5′- CGTTCTTCCCTCCCTTTTCCTTTCC-3′
IKB-α	Forward:5′-GAATCACCAGAACATCGTGAAG-3′
	Reverse: 5′- CAGTACTCCATGATTAGCACCT-3′
TNF-α	Forward:5′- CTCA TTCCTGCTTGTGGC −3′
	Reverse: 5′- CACTTGGTGGTTTGCTACG −3′
IL-1β	Forward: 5′- TTCCCA TTAGACAACTGC-3′
	Reverse: 5′- CTGTAGTGTTGTA TGTGA TC −3′
IL-6	Forward: 5′- CAGAACCGCAGTGAAGAG −3′
	Reverse: 5′- CAGAACCGCAGTGAAGAG −3′
IL-10	Forward: 5′- CAGAGCCAAAGCAGTGAGC −3′
	Reverse: 5′- TGACCCAGTCCATCCAGAG −3′
GRP78	Forward: 5′-GTCAGGGAGAGGAGGAAT-3′
	Reverse: 5′-TGGTGTCACTTATGGTAGAA-3′
IRE1	Forward:5′-TTGAAGTGGACAGTGAAGG-3′
	Reverse: 5′-TTGAAGTGGACAGTGAAGG-3′
PERK	Forward: 5′-GTAGCCACGACCTTCATC-3′
	Reverse: 5′-GTAGCCACGACCTTCATC-3′
ATF6	Forward: 5′-TGCCTTGGGAGTCAGACCTATGG-3′
	Reverse: 5′-CTGTGGACCGAGGAGAGGAGATG-3′
EIF2α	Forward: 5′-TGGTGGTTATCCGTGTTG-3′
	Reverse: 5′-CCGATTGCTTGAAGATGTC-3′
xBP1	Forward: 5′-TTGGGCATTCTGGACAAGTTGGAC-3′
	Reverse: 5′-AAAGGGAGGCTGGTAAGGAACTAGG-3′
ATF4	Forward: 5′-TCTGCCTTCTCCAGGTGGTTCC-3′
	Reverse: 5′-GCTGCTGTCTTGTTTTGCTCCATC-3′
CHOP	Forward: 5′-CTACTCTTGACCCTGCGTCCCTAG-3′
	Reverse: 5′-TCTTCCTTGCTCTTCCTCCTCTTCC-3′
Caspase8	Forward: 5′-CATCCTGACTGGCGTGAACTATGAC-3′
	Reverse: 5′-GTGAAGGTGGGCTGTGGCATC-3′
RIPK1	Forward: 5′-CGACTTCCAGACACCAAGCCATC-3′
	Reverse: 5′-TTTCCACTGCCTTCCCAGGTTTTC-3′
RIPK3	Forward: 5′-GAAGACACGGCACTCCTTGGTATC-3′
	Reverse: 5′-CTTGAGGCAGTAGTTCTTGGTGGTG-3′
MLKL	Forward: 5′-ACAGGCTACACCATTCGGAAACAC-3′
	Reverse: 5′-TCTGCTTTAGTGCTCTTTGCTGTCC-3′
Bax	Forward: 5′-GCTACAGGGTTTCATCCAGGATCG-3′
	Reverse: 5′-TGCTGTCCAGTTCATCTCCAATTCG-3′
Bcl-2	Forward: 5′-CCGTCGTGACTTCGCAGAGATG-3′
	Reverse: 5′-ATCCCTGAAGAGTTCCTCCACCAC-3′
CytC	Forward: 5′-AGGTGCCCGACTTCTCTGACTATC-3′
	Reverse: 5′-CCGCATAAGCAACACCCACAGTAG-3′
Caspase12	Forward: 5′-CCGTCCAGAGCACCAGTCCTC-3′
	Reverse: 5′-GCTTCACCCCACAGATTCCTTCC-3′
Caspase9	Forward: 5′-GCTCCAAGGACGACTTCATCAAGG-3′
	Reverse: 5′-CGCACTGCTCAGCTCACACTC-3′
Caspase3	Forward: 5′-ATGCTGCTCCCTTCCTCTTCCTC-3′
	Reverse: 5′-CACTTGTGTCTGTTGTTGCTGCTG-3′
MLC	Forward:5′-GATAGCCATCAGCAGCCTCACATC-3′
	Reverse: 5′- GCAACAGGAGCAGCAGGAGAAC-3′
RhoA	Forward:5′-ACGGTGTTTGAAAACTATGTGG-3′
	Reverse: 5′- GACAGAAATGCTTGACTTCTGG-3′
ROCK	Forward: 5′-GGGTGGTAGACTGGAGGGTTGG-3′
	Reverse: 5′-GGTAGGGTTTCTTCTGGGCTTTCTG-3′
CaM	Forward:5′-ACAAGGATGGGAATGGTTACAT-3′
	Reverse: 5′- TGCAGTCATCATCTGTACGAAT-3′
MLCK	Forward:5′-GGGCTGCCTCTCATCATCAATACG-3′
	Reverse: 5′- TGGATTCTGCTTCTGTGGGTAGGG-3′
β-actin	Forward: 5′-AATCCTGCGGCATCCACGAAAC-3′
	Reverse: 5′-CAGCACCGTGTTGGCGTAGAG-3′

### Intestinal flora staining

We collected 0.5 g of mouse small intestinal contents from each group and extracted the total DNA by the SDS-CTAB method. The purity and concentration of each set of DNA were tested using 2% agarose gel electrophoresis to see if the extracted DNA was acceptable. The qualified DNA product was diluted to 1 ng/μL and then subjected to PCR amplification in the V3-V4 region of the 16S rRNA gene. To ensure the amplification quality of PCR, use New England Biolabs Phusion High Fidelity PCR Master Mix with GC Buffer and High Fidelity enzyme. The recovered PCR amplification products were detected again by 2% agarose gel electrophoresis, and the qualified PCR products were purified by magnetic beads, and after the fluorescence quantification, the concentration of the PCR products was mixed in equal amounts, and the 2% agarose gel electrophoresis was detected again. Target strip recycling uses Qiagen’s gel recovery kit. Library construction uses the TruSeq DNA PCR-free Sample Preparation Kit. The constructed library quantification was performed using Qubit and Q-PCR. The NovaSeq6000 performs machine sequencing. The data were collected and analyzed using Qiime1 software. This experiment was commissioned by Novogene Tianjin Company.

### Western blotting

Total protein was extracted from intestinal tissue (0.1 mg) stored in an −80℃ refrigerator. Protein concentration was determined using the BCA method, leveled, and subjected to 80 V electrophoresis for 2 h. Subsequently, a 250-mA transfer of the membrane was performed based on different protein molecular weight sizes, and the membrane was closed for 2 h. The primary antibody was incubated overnight, followed by three washes with TBST. Next, the secondary antibody goat anti-rabbit IgG (1:2,000, Cell Signaling Technology) was added and incubated at room temperature for 1 h. Finally, the processed sample was developed and photographed using the SHST imaging system. Antibodies information for western blot is shown in Table 2.

**TABLE 2:** Antibodies required for western blot

Name	Cat no.	Company	Dilution times
β-actin	AC026	ABclonal Technology	1:1,000
GADPH	D16H11	Cell Signaling Technology	1:1,000
ZEB	70,512T	Cell Signaling Technology	1:1,000
E-cadherin	60335-1-Ig	Proteintech	1:2,000
ZO-1	13,663T	Cell Signaling Technology	1:1,000
GRP78	3,177S	Cell Signaling Technology	1:1,000
ATF6	WL01153	WanLei Bio	1:500
PERK	20582-1-AP	Proteintech	1:1,000
IRE1	27528-1-AP	Proteintech	1:1,000
CHOP	15204-1-AP	Proteintech	1:1,000
Caspase8	WL03426	WanLei Bio	1:1,500
p-RIPK1	53,286T	Cell Signaling Technology	1:1,000
p-RIPK3	91,702T	Cell Signaling Technology	1:1,000
p-MLKL	37,333T	Cell Signaling Technology	1:1,000
Bax	WL01637	WanLei Bio	1:500
Bcl-2	WL01556	WanLei Bio	1:500
Caspase12	WL03268	WanLei Bio	1:1,000
Caspase9	WL01551	WanLei Bio	1:500
Caspase3	WL04004	WanLei Bio	1:500
p-MLC	AF8618	Affinity	1:500
RhoA	10749-1-AP	Proteintech	1:1,000
ROCK	21850-1-AP	Proteintech	1:1,000

### ELISA

Each group weighed 0.1 g tissue samples and placed them into pre-cooled 9 mL PBS to grind the homogenate. The samples were then centrifuged at 13,000 rpm for 20 min at 4°C and the supernatants were collected for measurement of exosomal proteins IL-6, IL-10, IL-1β, and TNF-α. Each step was conducted strictly following the instructions of the ELISA kits (Nanjing Jiancheng BIO, Inc., China).

### Statistical analysis

SPSS 22.0 statistical software was used for statistical analysis. All data were expressed as ±standard deviation (SD) measurements. The data were compared using the *t*-test method. The significance of the different criteria was *P* < 0.05.

## Data Availability

The data sets supporting the conclusions of this article are available in the NCBI SRA under BioProject accession number PRJNA911678.
